# Influence of coincident distal radius fracture in patients with hip fracture: single-centre series and meta-analysis

**DOI:** 10.1007/s10195-013-0281-8

**Published:** 2013-12-29

**Authors:** C. E. Uzoigwe, M. Venkatesan, N. Johnson, K. Lee, S. Magaji, L. Cutler

**Affiliations:** Department of Trauma and Orthopaedics, University Hospitals of Leicester, Infirmary Square, Leicester, LE1 5WW UK

**Keywords:** Concomitant fracture, Hip, Wrist, Distal radius, Fracture, Mortality, Outcome

## Abstract

**Background:**

Hip and wrist fractures are the most common orthopaedic injuries. Combined hip and distal radius fractures are an important clinical and public health problem, since mobilisation and rehabilitation is challenging and likely to be prolonged in this setting. Few studies have explored the influence of an associated wrist fracture in patients with hip fracture. We present the largest series of patients with concomitant hip and wrist fractures. We perform the first meta-analysis of the literature on patients with concurrent hip and wrist fractures.

**Material and methods:**

In this single-centre retrospective study we compared 88 consecutive patients with simultaneous hip and wrist fractures with 772 consecutive patients who suffered isolated hip fractures.

**Results:**

Patients with the combined fracture were of a similar age compared to those with isolated hip fracture. There were a significantly higher proportion of women in the cohort with both hip and wrist fractures (female:male ratio of 9:1 versus 4:1 *p* < 0.0001). The combination fracture group had a greater length of hospitalisation (18 vs 13 days *p* < 0.0001). The survivorship of both groups was not significantly different even after adjustment for age and gender. Meta-analysis of the literature showed female preponderance, increased length of stay but no significant difference in survival in patients with concomitant hip and wrist fractures.

**Conclusion:**

The combination fracture occurs much more commonly in women and patients require a greater length of hospitalisation. The patients who sustained simultaneous hip and wrist fractures experienced no statistically significant difference in survivorship when compared to those who suffer isolated hip fractures. This is not withstanding the presence of two fractures. This difference in mortality did not reach statistical significance.

**Level of evidence:**

Level III (retrospective comparative study).

## Introduction

In England and Wales, the National Institute for Health and Clinical Excellence guidance on the management of patients with hip fractures reflects the ascendancy this injury has achieved in recent years over other injuries [1]. The hip fracture is arguably the most clinically significant fracture treated by the orthopaedic surgeon, given the high mortality associated with the injury. The wrist fracture is one of the most common fractures treated by the orthopaedic community. Few studies have explored the outcome of wrist fractures associated with neck of femur fractures. The purpose of the study is to compare the mortality of patients who sustain simultaneous hip and distal radius fractures to those who suffer isolated hip fractures.

## Materials and methods

### Clinical study

We identified all patients presenting to our unit with concomitant hip and wrist fractures between July 2004 and April 2011. We recorded the length of stay and date of mortality. We collected and compared similar data on patients presenting to our institution with isolated hip fracture between January 2010 and December 2010. Demographic data and information relating to the injury was collected, including the laterality of fracture for both groups. Our analysis looked retrospectively at outcomes for a large cohort of patients treated.

Normality testing was performed with the Shapiro–Wilk test. Normally distributed data was compared with the two-sample *t*-test. Non-parametric data underwent analysis with the Mann–Whitney *U* test. Nominal data was compared using Fisher’s exact test. The 95 % confidence interval for proportions was calculated using standard methods. Cox’s proportional hazards ratio was used to determine the effect of concomitant wrist fracture in patients with hip fracture while adjusting for age and gender.

### Meta-analysis

A PubMed search was performed to conduct the meta-analysis. Search terms were the MeSH term (neck of femur fracture) in combination with “wrist”, “upper limb” or “distal radius”. An iterative process was then used with the papers identified and their references. Meta-analysis was performed by pooling numerical data. When values were heterogeneous, in particular when combining data regarding early mortality, where some studies looked at in-hospital mortality and others recorded 30-day mortality, the random effects model was used.

## Results

### Clinical study

Of the 5,164 patients presenting to our unit with hip fracture between July 2004 and April 2011, we identified 88 patients with concomitant hip and distal radius fractures. The injuries were ipsilateral in 91 % (80) and contralateral in only 9 % (8). There were 9 men and 79 (89 %) women. The mean age was 79 years (range 26–99). Most (16 %) of the patients with combined hip fractures presented in 2010.

The control group thus consisted of all the patients presenting to our unit from January 2010 to December 2010. Seven hundred and seventy-two patients with isolated hip fractures were identified presenting to our unit in the relevant time frame (January 2010–December 2010). There were 532 (69 %) women and 240 men. The mean age was 80 years (range 22–105). There was a statistically significant difference in gender distribution between the isolated hip fracture and combination hip and wrist fracture cohorts (Table 1). There was a female preponderance in both groups. This was much more marked in the cohort with concomitant wrist fractures (*p* < 0.0001). The 30-day and 1-year mortality was not significantly different for the combined fracture group compared to the isolated fracture group. Those with concurrent fractures had a longer in-hospital stay compared to those with isolated hip fractures (median 18 vs 13 days *p* < 0.0001) (Table 1).

**Table 1:** Comparison of patients with isolated fracture and combination hip and wrist fractures

	Isolated hip fracture	Hip and wrist fracture	*p* value
Age	80	79	0.45
Female:male ratio	9:1	4:1	<0.0001
30-day mortality (%)	9.6	9.1	0.33
90-day mortality	18	16	0.66
1-year mortality (%)	31	25	0.33
Median length of stay	13	18	<0.001

**Fig. 1:**
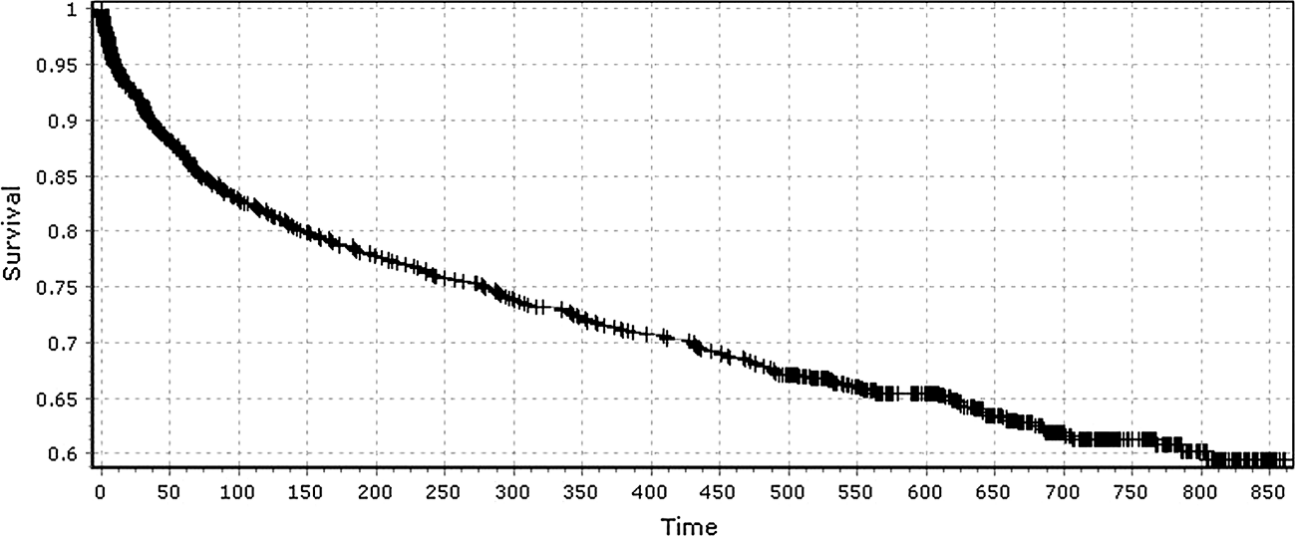
Survivorship curve of patients with isolated hip fracture

**Fig. 2:**
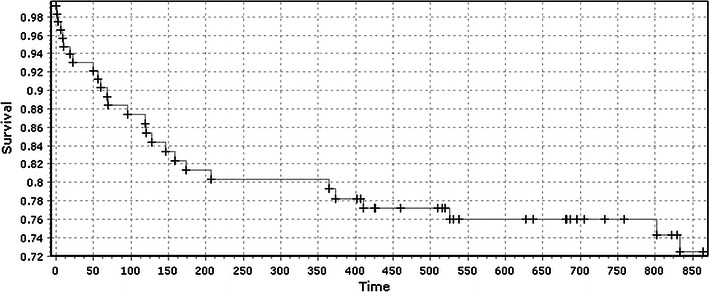
Survivorship curve of patients with combined hip and wrist fracture

Cox’s proportional hazard analysis, adjusting for gender and age, showed that the presence of a concomitant wrist fracture did not significantly affect mortality (*p* = 0.45) (Table 2). Age and gender were strong predictors of survivorship. Female sex and youth were associated with improved survivorship. The risk ratio of 1.05 for age means that for every year increase in age, the odds of death at any given time increase by a factor of 1.05. Similarly, as far as gender is concerned, the odds of death for women at any given time is 0.54 that for men. The survivorship curves for patients with isolated hip fracture and the combination hip and wrist fracture had a similar profile (Figs. 1, 2).

**Table 2:** Cox regression analysis for patients with hip fracture examining the effect of age, gender and concurrent wrist fracture

Covariate	Risk ratio	Lower 95 % CI	Upper 95 % CI	*p* value
Age	1.05	1.04	1.06	<0.0001
Gender	0.54	0.42	0.68	<0.0001
Concomitant wrist fracture	0.86	0.57	1.28	0.45

*CI* confidence interval

There was no statistical significance between the groups in the method of fixation of hip fractures of the two groups (Table 3).

**Table 3:** Methods of fixation of hip fracture in patients with isolated hip fracture and combined hip and wrist fracture

	Isolated hip fracture	Hip and wrist fracture
Hemiarthroplasty	350 (45 %)	41 (47 %)
Nail	99 (13 %)	15 (17 %)
Dynamic screw	276 (36 %)	28 (32 %)
Cannulated screw	19 (2 %)	1 (1 %)

### Meta-analysis

**Fig. 3:**
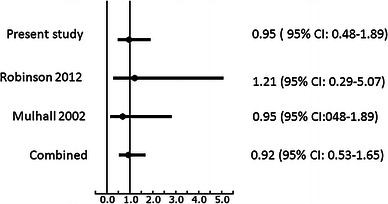
Forest plot of studies exploring the effect of concomitant wrist fracture on early mortality in patients with hip fracture. Early mortality refers to in-hospital or 30-day mortality

Four studies matched the search criteria (Mulhall et al. [2], Shabat et al. [3], Tow et al. [4], Robinson et al. [5]). Two percent (95 % CI 1.7–2.4) of patients with hip fracture suffered a concurrent wrist fracture. Pooling our data with that of Robinson et al., the presence of a wrist fracture did not adversely affect risk of death at 1 year. Performing a random effects meta-analysis, given the slightly different time frames, of the three studies (Robinson et al. [5], Mulhall et al. [2]), we found that the presence of a wrist fracture with hip fracture resulted in no difference in survivorship in the short term (namely 30-day or in-hospital mortality). This is confirmed in the forest plot (Fig. 3).

## Discussion

Wrist and hip fractures are two of the most common and clinically significant fractures treated by the orthopaedic surgeon. When they occur in combination they pose a peculiar challenge. Expeditious surgery is mandated in hip fracture patients as it is thought to improve survivorship [6]. In England and Wales, the Department of Health provides financial incentives, in the form of the Best Practice Tariff, to National Health Service Trusts where patient care meets a minimum standard [7]. Surgery within 36 h is one criterion to be satisfied if trusts are to enjoy the Tariff. Functionality and independence are dependent upon hip and wrist function. No previous studies have examined the effect of concomitant wrist fractures in hip fracture patients, correcting for potential confounders.

In the present study the combination and singular fracture cohorts were recruited from different time frames. This was necessary to include sufficient numbers of patients with hip and distal radius fractures. This may potentially impact upon our findings. Over the periods in question (2004–2011 for hip/wrist and 2010 for hip fracture) operative fixation remained the mainstay of treatment for hip fractures. In particular, there was no significant difference in the method of fracture fixation for the two cohorts (Table 3).

Those with combination hip and wrist fractures did not have a significantly different 30-day, 90-day and 1-year mortality compared to those with isolated hip fractures. There was a considerably higher proportion of women in the combination fracture group (89 vs 69 %, respectively *p* < 0.0001). Correcting for both age and gender with Cox’s proportional hazard regression analysis, we found that the presence of an associated wrist fracture did not significantly impact upon mortality in patients with hip fractures.

A review of the literature, involving smaller studies, suggests equally interesting findings (Table 4). In 2002 Mulhall [2] performed an analysis of all patients presenting to his institution with simultaneous hip and upper limb fractures. He found that wrist fractures were the most common upper limb fracture associated with hip fractures. He also observed a significant female preponderance when compared to patients with isolated hip fractures. The combination fracture cohort had a longer in-hospital stay and lower in-hospital mortality.

**Table 4:** Meta-analysis of studies exploring effect of coincident hip fracture in patients with hip and wrist fracture

Study	Number of patients	Prevalence of hip fracture with wrist fracture	Mean age isolated hip vs combined fracture	Female: male isolated hip vs combined fracture	30-day mortality isolated hip vs combined fracture	1-year mortality isolated hip vs combined fracture	Length of stay isolated hip vs combined fracture (median)	Adjusted mortality ratio isolated hip vs combined fracture
Mulhall et al. [2]	28	3.7 %	77 vs 84*	3:1 vs 8:1*	10.3 vs 5.6 % (in-hospital mortality)*		15.6 vs 20.4 (mean)	
Tow et al. [4]	33	2.6 %	78 vs 79	2:1 vs 6:1			17 vs 23	
Robinson et al. [5]	34	1.8 %	82 vs 83	4:1 vs 7:1	6.4 vs 7.7 %	28 vs 19 %	13 vs 17.5	
Shabat et al. [3]	46			7:1 (no data of isolated hip fracture)			13 (no data of isolated hip fracture)	
This study	88	1.7 %	80 vs 79	4:1 vs 9:1	9.6 vs 9.1 %	30.6 vs 25 %	13 vs 18	0.86 (95 % CI 0.57–1.28)
Meta-analysis	229	2.0 (95 % CI 1.7–2.4)	79.8 vs 80.5	3:1 vs 7:1 (*p* < 0.0001)	Relative risk 0.93 (95 % CI 0.53–1.65)	29 vs 24 % (*p* = 0.2) relative risk 0.81 (95 % CI 0.58–1.13)		

* For all patients with hip and upper limb fractures

Tow et al. [4] performed a matched case–control study. In this they compared 33 patients with coincident hip and wrist fractures with 33 patients suffering from isolated hip fractures. The comparators were matched for age and gender. They observed a similar female predilection. Tow et al. interestingly observed that the combination fracture group were slightly more osteoporotic than those in the isolated hip fracture group, but the difference was not statistically significant.

Robinson and co-workers, in 2012, analysed the features of patients with concomitant hip and upper limb fractures. Similar to Mulhall they observed distal radius fractures to be the most common associated injury [5]. Consistent with our study and preceding works, Robinson noted that there was a high female:male ratio and longer length of hospital stay, in instances of hip and concomitant wrist fracture.

Pooling the available data from the literature, we observed that 2 % (95 % CI 1.7–2.4) of patients with hip fracture suffered a concurrent wrist fracture. The narrow confidence interval suggests the accuracy of the value. Both cohorts had a similar age. All previous studies found a much higher proportion of female patients in the group with combined wrist and hip fracture. We considered whether the similar survivorship observed in patients with simultaneous hip and wrist fractures, in spite of the presence of two fractures, was due to the female preponderance acting as a confounder. Male patients have a much higher mortality following hip fracture compared with women. The most recent meta-analysis, involving in excess of 64,000 patients, indicates that male sex engenders a 1.7-fold increase in mortality compared to female patients [8]. We thus decided to adjust mortality for gender and age. In this present study, using Cox’s proportional hazard analysis adjusted for age and gender, there remained a non-significant difference in survivorship in patients with hip and wrist fractures compared to those with isolated wrist fractures. No adjustment was made for potential differences in co-morbidities between the two cohorts. However, both samples were sufficiently large to be representative and correction was made for the pre-eminent difference, namely gender. Further, differences in the mortality between male and female hip fracture patients are not related to co-morbid status [9].

A minority of patients with hip fractures sustain concomitant wrist fractures. However, given the incidence of hip fractures, this number is not negligible. This is the largest study exploring the outcome of concomitant hip and wrist fractures. This is the first meta-analysis of studies examining the natural history of patients with synchronous hip and wrist fractures. The combination fracture occurs much more commonly in women and patients require a greater length of hospitalisation. The patients who sustained simultaneous hip and wrist fractures suffered no significant difference in survivorship when compared to those who suffer isolated hip fractures. It is tempting to assume that the combination fracture is indicative of a frailer patient and poses a greater risk to life. However, our findings and the meta-analysis suggest that the combination hip and wrist fracture does not portend increased mortality compared to patients with isolated hip fractures.
